# Data on dysfunctional muscle contraction and genes contractile expression associated with chlorpyrifos exposure in slow twitch skeletal muscle^☆^

**DOI:** 10.1016/j.dib.2019.104775

**Published:** 2019-11-09

**Authors:** Nancy Hallal, Mahmoud Khalil, Mohamed E. Moustafa, Wiam Ramadan, Wissam H. Joumaa

**Affiliations:** a Department of Biological Sciences, Faculty of Science, Beirut Arab University, Lebanon; b Rammal Hassan Rammal Research Laboratory, PhyToxE Research Group, Faculty of Sciences, Lebanese University, Nabatieh, Lebanon; c Department of Biochemistry, Faculty of Sciences, Alexandria University, Egypt; d Lebanese Institute for Biomedical Research and Application (LIBRA), Lebanese International University (LIU), Beirut, Lebanon

**Keywords:** Acetylcholinesterase, Chlorpyrifos, Contractility, Nicotinic acetylcholine receptor, Ryanodine receptor, Sarco/endoplasmic reticulum Ca2^+^-ATPase, Soleus

## Abstract

Chlorpyrifos (CPF) is a toxic organophosphate commonly used worldwide. Its residues are being detected in different environmental matrixes and hence in the food chain. Repeated CPF exposure might pose health risk for the general population on long term. This data article contains the data of contractility impairment further to dietary exposure to CPF on a hind limb skeletal muscle; soleus, a typical slow twitch skeletal muscle. Thirty adult male rats Sprague Dawley are divided into three groups receiving the following daily diet for 6 weeks: Group 1 (vehicle), Group 2: CPF1 (CPF 1mg/kg/day) and Group 3: CPF5 (CPF 5 mg/kg/day). Soleus twitch tension and fatigability index are determined at the end of the treatment. The activity of acteylcholinesterase enzyme is assessed in the tissues homogenate. Additionally, we examined the expression levels of ryanodine type 1 receptor (RyR1), ATPase Sarcoplasmic/Endoplasmic Reticulum Ca^2+^ Transporting 1 (Atp2a1), ATPase Sarcoplasmic/Endoplasmic Reticulum Ca^2+^ Transporting 2 (Atp2a2) and nicotinic acetylcholine receptor (nAChR) in CPF-exposed skeletal muscle tissue using quantitative real time polymerase chain reaction.

CPF exposure at two different doses induced an increase in twitch contraction in soleus muscle along with an increase in fatigability index. These increases are accompanied by low level of acetylcholinesterase enzyme activity as well as modification in genes level expression of nAChR, RyR1, Atp2a1 and Atp2a2 involved in contractility.

**Table:** Specifications Table

Subject	Biology
Specific subject area	Physiology, toxicology, Molecular biology
Type of data	Graph
How data were acquired	Physiology (PIODEN CONTROLS LTD, United Kingdom); Data acquisition system (Digidata 1200, Axon Instruments); spectrophotometry (microplate reader; MR-96A, Mindray); quantitative real time PCR (q-RT-PCR) (CFX96 system thermal cycler (Bio Rad Laboratories, USA)
Data format	Raw and Analysed
Parameters for data collection	Rats are fed daily a diet mixed with chlorpyrifos at 1 or 5 mg/kg for six consecutive weeks.
Description of data collection	Twitch contraction and fatigability of soleus muscle are determined *in vitro* after 6 weeks of chlorpyrifos exposure. Acetylcholineesterase enzyme activity is measured spectrophotometrically in tissues homogenate and genes expression analysis is done by q-RT-PCR.
Data source location	Rammal Hassan Rammal Research Laboratory, PhyToxE research group, Faculty of Sciences (V), Lebanese University. Nabatieh Lebanon
Related research article	Nancy Hallal, Hiba El Khayat El Sabbouri, Ali Salami, Wiam Ramadan, Hassan Khachfe, Mohamed E. Moustafa, Mahmoud Khalil, Wissam H. Joumaa Impacts of prolonged chlorpyrifos exposure on locomotion and slow-and fast- twitch skeletal muscles contractility in rats. toxicology reports https://doi.org/10.1016/j.toxrep.2019.06.006

**Table:** 

**Value of the Data** • The data reveals the impairment of soleus contractions in CPF-treated rats at two doses which may be interesting for researchers investigating the physiology of skeletal muscles further to organophosphate intoxication. • The data reveals the expression levels of some genes involved in muscle contraction and responsible of calcium flux. The data may be useful in suggesting additional mechanism of action for CPF other than the acetlycholinesterase inhibition. • The data provides the basis for deeper investigation to understand the modification of the contractile machinery of skeletal muscle further to subchronic intoxication with CPF.

## 1 Data

### 1.1 Effect of CPF on soleus twitch tension

*S*oleus twitch tension properties are recorded the day of the sacrifice (week 6) for the 3 groups. Twitch tension in soleus control group is characterized by an amplitude of 104.75 ± 4.84 g/cm^2^, contraction time of 87.13 ± 6.21 ms and half relaxation time of 99.32 ± 4.40 ms (n = 10). A significant increase in the amplitude of twitch tension by 28% and 67% in CPF1 and CPF5 groups is recorded respectively (p < 0.05). The other parameters, contraction time and half relaxation time are not significantly modified in exposed rats compared to control.

### 1.2 Effect of CPF on soleus fatigability index

**Fig. 1:**
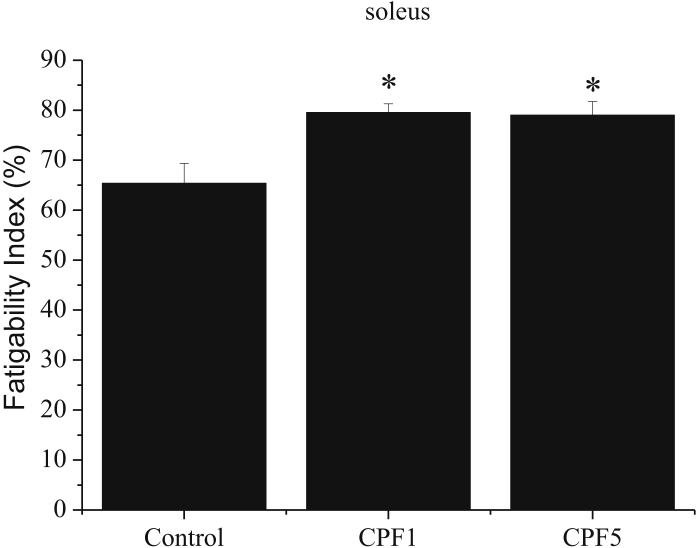
Effect of CPF exposure on soleus fatigability index. Data are quoted as the means ± S.E.M. *p < 0.05, n = 10.

A significant effect of CPF exposure on fatigability index is observed in soleus muscle. The fatigability index is higher by 21% in CPF1 and CPF5 compared to the control group (p < 0.05) (Fig. 1). There is no dose related difference in soleus fatigability index.

### 1.3 Effect of CPF on AChE activity

**Fig. 2:**
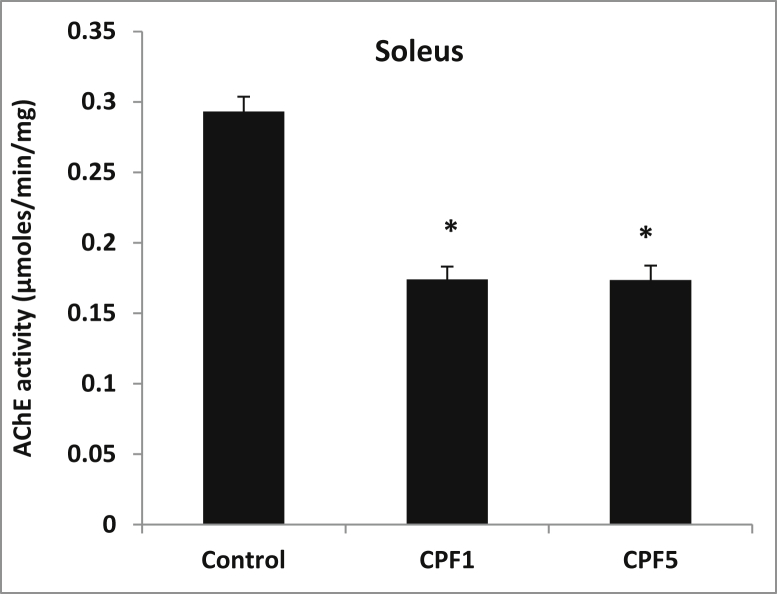
Effect of CPF exposure on AChE activity in soleus. Data are quoted as the means ± S.E.M. *p < 0.05, n = 10.

AChE activity data revealed that CPF inhibited the enzyme activity in soleus of treated rats when compared to control group. The inhibition of AChE was about 41% in the two doses tested CPF1 and CPF5. (p < 0.05) (Fig. 2).

### 1.4 Real-time PCR detection for nAChR, RyR1, Atp2a1 and Atp2a2 mRNA expression

**Fig. 3:**
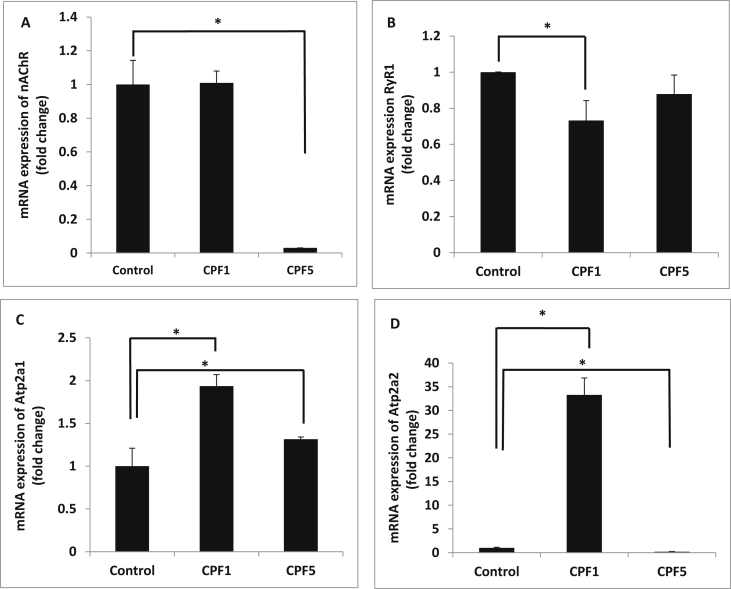
Contractile machinery genes expression: A (nAChR), B (RyR1), C (Atp2a1), D (Atp2a2), in CPF1 and CPF5 treated soleus as evaluated by q-RT-PCR. Data are expressed relative to gene expression in controls, according to the 2^−ΔΔCT^ method (quoted as the mean ± SEM). *p < 0.05.

A significant decrease is observed in mRNA expression levels of nAChR and RyR1 after CPF5 and CPF1 treatments respectively. On the other hand, a significant increase is detected in mRNA expression levels of Atp2a1 after CPF1 and CPF5 treatments and Atp2a2 at CPF1 treatment.(See Fig. 3). The raw data related to Fig. 1, Fig. 2 and Fig. 3 were shown in Appendix A. supplementary data.

## 2 Experimental design, materials, and methods

### 2.1 Animals’ treatment

Animal use protocol is approved and performed in accordance with the guidelines for animal care issued by the institutional review board (IRB) at Beirut Arab University (Approval code:2019H-0036-S-P-0295).

Male Sprague Dawley rats, approximately weighing 200 g at the beginning of the study, were habituated for one week to the local conditions (temperature 23 °C, 12 h:12h light/darkness cycle) prior to the start of the experimentation. The rats were divided randomly into three groups. Control, CPF1 and CPF5 groups received daily standardized diet mixed with corn oil only, 1 mg/kg of CPF and 5 mg/kg of CPF respectively for six consecutive weeks. Each experimental group had free access to water. CPF (O, O′ - diethyl- O- 3, 5, 6- trichloro- 2-pyridylphosphorothioate) was purchased from Sigma –Aldrich and dissolved in corn oil.

### 2.2 Animals sacrifice and muscles dissection

After six weeks of exposure, the animals were euthanized with an intraperitoneal overdose of sodium pentobarbital (1 ml/kg; 200 mg/ml solution) and sacrificed. *S*oleus was immediately collected from both hind limbs. One soleus was deep frozen and stored at −80 °C for acetylcholinesterase activity assay and genes expression level. The other soleus dissected was examined immediately for its contractility parameters.

### 2.3 Soleus contractility and fatigue resistance

The entire soleus dissected was directly transferred to oxygenated room filled with mammalian physiological solution (140 mM NaCl, 6 mM KCl, 5 mM HEPES, 3 mM CaCl_2_ adjusted to pH 7.35). It was then appended at the distal and proximal tendons to a force transducer, stretched to optimal length (L_0_), and electrically stimulated (12 V, 2 Hz for 5 minutes) through platinum electrodes positioned near the muscle. Contractions were recorded using a bridge amplifier and data acquisition system (Digidata 1200, Axon Instruments) controlled by custom-made software.

The peaks twitch tension (g/cm^2^), contraction time (ms), half-relaxation time (ms) were determined. Force after 5 minutes of continuous stimulation was calculated as percentage of maximum force and was termed fatigability index (%) as described elsewhere [2].

### 2.4 Acetylcholinesterase activity

Acetylcholinestarase assay kit (ab138871, abcam, UK) was used for the detection of acetylcholinesterase activity. The kit provided a colorimetric one step assay for the detection of acetylcholinesterase activity by applying a modified Ellman method [3]. The procedure was done according to the manufacturer's instructions. Briefly, 20 mg of soleus were washed with cold phosphate buffer saline and homogenized with mammalian cell lysis buffer 5× (ab179835, abcam, UK). The homogenates were centrifuged at 2500 rpm for 10 min. and the supernatant was used for acetylcholinesterase assay. 50 μl of the crude enzyme extracts were added to 96 well plates containing 50 μl of the reaction mixture (20 × 5, 5′ dithio-bis-2-nitrobenzoic acid and 20× acetylthiocholine). The absorbance was measured spectrophotometrically at 414 nm with a microplate reader (MR-96A, Mindray). The protein concentrations of the isolated acetylcholinestrase were measured spectrophotometrically according to Bradford protein assay with bovine serum albumin as standard.

### 2.5 Determination of nAChR, RyR1, Atp2a1 and Atp2a2 mRNA levels using q-RT- PCR

Total RNA was isolated from soleus using Quick-RNA MiniPrep Plus kit (Zymo Research, USA) according to the manufacturer's instructions. RNA concentration was determined in a Qubit 3.0 fluorometer using the Qubit™ RNA HS Assay Kit (Thermo Fisher Scientific, Malaysia). 1 μg of RNA was reverse transcribed to cDNA by iScript™ cDNA Synthesis Kit and then amplified by qPCR using iTaq™ Universal SYBR® Green Supermix in a CFX96 system (Bio Rad Laboratories, USA). GADPH was used as the reference gene. Primers were purchased from Macrogen, Korea. The sequences of the primer pairs were as follow:

<nAChR> forward: 5′- TGT GTC TCA TCG GGA CGC-3′ reverse: 5′- GGG CAG AGG GAG GCT TAG TTC -3′

<RyR1> forward: 5′-ATC CTT TCA TCC GTC ACT C-3′ reverse: 5′-CAG GCT CGT CCT CAT CTT-3′

< Atp2a1> forward: 5′-CCT ACA CTG GCC GTG AGT TT-3′ reverse: 5′-AGA GCA GGG GCA TCA TTG AC-3′

< Atp2a2> forward: 5′-TCA CAC CGC TGA ATC TGA CC-3′ reverse: 5′-ACT CCA GTA TTG CAG GCT CC-3′

<GADPH> forward: 5′-CAG GGC TGC CTT CTC TTG TG-3′ reverse: 5′-AAC TTG CCG TGG GTA GAG TC-3′

All PCR reactions were performed in triplicate. Data, expressed as relative fold change in studied gene expression, was calculated using 2 ^-ΔΔCq^ after being normalized to reference gene GADPH.

## 3 Data analysis

The data are expressed as the means ± S.E.M. Statistical comparisons using one-way analysis of variance (ANOVA) were used to determine statistical significance. The significance level for the main treatment effects was set at p < 0.05.
