# Supplementary material for: Data on dysfunctional muscle contraction and genes contractile expression associated with chlorpyrifos exposure in slow twitch skeletal muscle
Source: Data Brief. 2019 Nov 9;27:104775. doi: 10.1016/j.dib.2019.104775 (PMC6920330; doi:10.1016/j.dib.2019.104775)
Supplement: Multimedia component 1 [file mmc1.pdf]

Contractility Parameters Raw data (Twitch)								
Amplitude (g/cm^2)			Time to Peak Tension (ms)			½ time of relaxation (ms)		
Control	CPF1	CPF5	Control	CPF1	CPF5	Control	CPF1	CPF5
117.77	145.32	136.00	85.13	66.96	120.54	106.84	75.89	105.36
90.05	162.85	203.14	103.04	75.89	111.61	105.65	98.21	111.61
83.66	163.87	190.20	74.68	111.61	111.61	112.39	107.14	116.07
124.35	126.97	122.44	102.77	107.14	109.82	97.19	107.14	102.68
105.42	90.77	162.31	110.33	80.36	58.39	86.71	93.75	60.48
89.42	134.33	145.6	109.71	120.54	64.52	114.90	116.07	75.00
114.21	139.91	207.68	66.68	60.32	74.84	80.68	94.60	80.48
125.95	118.30	215.12	63.42	58.87	86.13	79.21	117.70	84.19
101.81	144.69	170.48	60.16	80.65	72.58	93.50	90.51	105.51
94.90	120.20	200.70	95.41	65.97	92.42	116.1	104.61	102.56

Fatigability Index								
CONTROL			CPF1			CPF5		
Initial Amplitude (mV)	End Amplitude (mV)	FI (%)	Initial Amplitude (mV)	End Amplitude (mV)	FI (%)	Initial Amplitude (mV)	End Amplitude (mV)	FI (%)
1300	418.75	67.79	1300	300	76.92	4956.25	1200	75.79
943.75	287.5	69.54	437.5	75	82.86	5500	1093.75	80.11
318.75	125	60.78	3550	575	83.80	987.5	175	82.28
1237.5	471.875	61.87	2975	650	78.15	3012.5	587.5	80.50
1262.5	428.75	66.04	400	85.625	78.59	2562	499	80.52
5000	1662.5	66.75	3681.25	600	83.70	1270.5	321	74.73
800	293.75	63.28	4228	1005	76.23	1744.5	329	81.14
862.5	260	69.86	3794.5	840	77.86	3371.5	556	83.51
725	245	66.21	3122.5	535	82.87	5392.5	1228	77.23
950	325	65.79	4935	1020	79.33	2777.5	690	75.16

AchE Activity (µmol/min/mg) (Avera		
Control	CPF1	CPF5
0.31436	0.17877	0.16649
0.27876	0.16984	0.17874
0.26946	0.16047	0.16948
0.3126	0.17369	0.16545
0.30579	0.17848	0.16854
0.29512	0.18168	0.17071
0.28413	0.17853	0.17511
0.30463	0.17991	0.17442
0.27312	0.17136	0.18626
0.29283	0.16518	0.17987

Trail 1:

TARGET	SAMPLE	group	Cq	expre	EXP STE	MEANCq	Cq SEM
RYR1	Soleus	Control	27.59	1	0.00194	27.68	0.05207
RYR1	Soleus	Control	27.77				
RYR1	Soleus	Control	27.69				
RYR1	Soleus	CPF1	28.87	0.7136	0.0211	29.05	0.10392
RYR1	Soleus	CPF1	29.23				
RYR1	Soleus	CPF1	29.05				
RYR1	Soleus	CPF5	28.12	0.852	0.2761	28.33	0.12143
RYR1	Soleus	CPF5	28.54				
RYR1	Soleus	CPF5	28.35				

TARGET	SAMPLE	group	Cq	MEAN
GADPH	Soleus	Control	20.79	21.04
GADPH	Soleus	Control	21.21	
GADPH	Soleus	Control	21.12	
GADPH	Soleus	CPF1	22.12	21.92
GADPH	Soleus	CPF1	21.72	
GADPH	Soleus	CPF1	21.92	
GADPH	Soleus	CPF5	21.2	21.46
GADPH	Soleus	CPF5	21.73	
GADPH	Soleus	CPF5	21.46	

Trail 2:

TARGET	SAMPLE	group	Cq	expre	EXP STE	MEANCq	Cq SEM
RYR1	Soleus	Control	27.4	1	0.00227	27.66	0.13
RYR1	Soleus	Control	27.79				
RYR1	Soleus	Control	27.79				
RYR1	Soleus	CPF1	29.13	0.72	0.161	29.03	0.04842
RYR1	Soleus	CPF1	28.98				
RYR1	Soleus	CPF1	28.99				
RYR1	Soleus	CPF5	29.01	0.827	0.216436	28.73	0.14836
RYR1	Soleus	CPF5	28.7				
RYR1	Soleus	CPF5	28.5				

TARGET	SAMPLE	group	Cq	MEAN
GADPH	Soleus	Control	21.2	21.01
GADPH	Soleus	Control	20.89	
GADPH	Soleus	Control	20.95	
GADPH	Soleus	CPF1	21.82	21.91
GADPH	Soleus	CPF1	22.12	
GADPH	Soleus	CPF1	21.8	
GADPH	Soleus	CPF5	21.88	21.81
GADPH	Soleus	CPF5	21.75	
GADPH	Soleus	CPF5	21.82	

Trail 3:

TARGET	SAMPLE	group	Cq	expre	EXP STE	MEANCq	Cq SEM
RYR1	Soleus	Control	27.86	1	0.00141	27.91	0.03844
RYR1	Soleus	Control	27.9				
RYR1	Soleus	Control	27.99				
RYR1	Soleus	CPF1	29.05	0.718	0.176	29.01	0.11667
RYR1	Soleus	CPF1	28.8				
RYR1	Soleus	CPF1	29.2				
RYR1	Soleus	CPF5	28.99	0.862	0.212	28.78	0.10837
RYR1	Soleus	CPF5	28.62				
RYR1	Soleus	CPF5	28.75				

TARGET	SAMPLE	group	Cq	MEAN
GADPH	Soleus	Control	21.3	21.16
GADPH	Soleus	Control	20.99	
GADPH	Soleus	Control	21.2	
GADPH	Soleus	CPF1	21.7	21.78
GADPH	Soleus	CPF1	21.82	
GADPH	Soleus	CPF1	21.84	
GADPH	Soleus	CPF5	21.63	21.82
GADPH	Soleus	CPF5	21.94	
GADPH	Soleus	CPF5	21.89	

Trial 1:

TARGET	SAMPLE	group	Cq	expre	EXP STE	MEANCq	Cq SEM
SERCA1	Soleus	Control	26.9	1	0.00075	26.87	0.02517
SERCA1	Soleus	Control	26.89				
SERCA1	Soleus	Control	26.82				
SERCA1	Soleus	CPF1	27.9	1.78	0.26	27.583	0.15962
SERCA1	Soleus	CPF1	27.46				
SERCA1	Soleus	CPF1	27.39				
SERCA1	Soleus	CPF5	27.82	1.33	0.14	27.71	0.05608
SERCA1	Soleus	CPF5	27.63				
SERCA1	Soleus	CPF5	27.69				

TARGET	SAMPLE	group	Cq	MEAN
GADPH	Soleus	Control	20.6	20.5
GADPH	Soleus	Control	20.47	
GADPH	Soleus	Control	20.45	
GADPH	Soleus	CPF1	21.9	22.05
GADPH	Soleus	CPF1	22.22	
GADPH	Soleus	CPF1	22.05	
GADPH	Soleus	CPF5	21.64	21.77
GADPH	Soleus	CPF5	21.92	
GADPH	Soleus	CPF5	21.75	

Trial 2:

TARGET	SAMPLE	group	Cq	expre	EXP STE	MEANCq	Cq SEM
SERCA1	Soleus	Control	27.01	1	0.00124	26.89	0.06429
SERCA1	Soleus	Control	26.79				
SERCA1	Soleus	Control	26.87				
SERCA1	Soleus	CPF1	27.51	1.98	0.19	27.39	0.05897
SERCA1	Soleus	CPF1	27.35				
SERCA1	Soleus	CPF1	27.32				
SERCA1	Soleus	CPF5	28.82	1.23	0.38	28.52	0.26577
SERCA1	Soleus	CPF5	28.75				
SERCA1	Soleus	CPF5	27.99				

TARGET	SAMPLE	group	Cq	MEAN
GADPH	Soleus	Control	20.69	20.57
GADPH	Soleus	Control	20.54	
GADPH	Soleus	Control	20.49	
GADPH	Soleus	CPF1	21.82	22.06
GADPH	Soleus	CPF1	22.24	
GADPH	Soleus	CPF1	22.12	
GADPH	Soleus	CPF5	22.49	22.51
GADPH	Soleus	CPF5	22.4	
GADPH	Soleus	CPF5	22.65	

Trial 3:

TARGET	SAMPLE	group	Cq	expre	EXP STE	MEANCq	Cq SEM
SERCA1	Soleus	Control	26.99	1	0.00109	26.88	0.07055
SERCA1	Soleus	Control	26.91				
SERCA1	Soleus	Control	26.75				
SERCA1	Soleus	CPF1	27.61	2.06	0.18	27.4	0.12423
SERCA1	Soleus	CPF1	27.41				
SERCA1	Soleus	CPF1	27.18				
SERCA1	Soleus	CPF5	27.82	1.4	0.09	27.78	0.03512
SERCA1	Soleus	CPF5	27.81				
SERCA1	Soleus	CPF5	27.71				

TARGET	SAMPLE	group	Cq	MEAN
GADPH	Soleus	Control	20.55	20.5
GADPH	Soleus	Control	20.51	
GADPH	Soleus	Control	20.44	
GADPH	Soleus	CPF1	22.05	22.06
GADPH	Soleus	CPF1	22.09	
GADPH	Soleus	CPF1	22.05	
GADPH	Soleus	CPF5	21.98	21.88
GADPH	Soleus	CPF5	21.87	
GADPH	Soleus	CPF5	21.81	

Trial 1:

TARGET	SAMPLE	TREAT	Cq	exp	EXP STE	MEANCq	Cq SEM
SERCA2	Soleus	Control	29.73	1	0.00085	29.65	0.04933
SERCA2	Soleus	Control	29.66				
SERCA2	Soleus	Control	29.56				
SERCA2	Soleus	CPF1	25.18	35.58	0.08	25.08	0.05812
SERCA2	Soleus	CPF1	24.98				
SERCA2	Soleus	CPF1	25.1				
SERCA2	Soleus	CPF5	30.84	0.2	0.11	30.73	0.05608
SERCA2	Soleus	CPF5	30.65				
SERCA2	Soleus	CPF5	30.71				

TARGET	SAMPLE	group	Cq	MEAN
GADPH	Soleus	Control	22.59	22.64
GADPH	Soleus	Control	22.71	
GADPH	Soleus	Control	22.63	
GADPH	Soleus	CPF1	23.23	23.23
GADPH	Soleus	CPF1	23.23	
GADPH	Soleus	CPF1	23.24	
GADPH	Soleus	CPF5	21.45	21.42
GADPH	Soleus	CPF5	21.33	
GADPH	Soleus	CPF5	21.5	

Trial 2:

TARGET	SAMPLE	TREAT	Cq	exp	EXP STE	MEANCq	Cq SEM
SERCA2	Soleus	Control	28.99	1	0.00191	28.74	0.13383
SERCA2	Soleus	Control	28.53				
SERCA2	Soleus	Control	28.71				
SERCA2	Soleus	CPF1	25.68	26.66	0.07	25.62	0.02963
SERCA2	Soleus	CPF1	25.61				
SERCA2	Soleus	CPF1	25.58				
SERCA2	Soleus	CPF5	31.14	0.16	0.06	31.18	0.02186
SERCA2	Soleus	CPF5	31.21				
SERCA2	Soleus	CPF5	31.2				

TARGET	SAMPLE	group	Cq	MEAN
GADPH	Soleus	Control	21.59	21.62
GADPH	Soleus	Control	21.63	
GADPH	Soleus	Control	21.64	
GADPH	Soleus	CPF1	23.32	23.236
GADPH	Soleus	CPF1	23.2	
GADPH	Soleus	CPF1	23.19	
GADPH	Soleus	CPF5	21.42	21.41
GADPH	Soleus	CPF5	21.47	
GADPH	Soleus	CPF5	21.35	

Trial 3:

TARGET	SAMPLE	TREAT	Cq	exp	EXP STE	MEANCq	Cq SEM
SERCA2	Soleus	Control	28.82	1	0.00112	28.75	0.03283
SERCA2	Soleus	Control	28.74				
SERCA2	Soleus	Control	28.71				
SERCA2	Soleus	CPF1	25.11	37.53	0.09	25.08	0.02848
SERCA2	Soleus	CPF1	25.12				
SERCA2	Soleus	CPF1	25.03				
SERCA2	Soleus	CPF5	30.24	0.25	0.19	30.2	0.13691
SERCA2	Soleus	CPF5	29.95				
SERCA2	Soleus	CPF5	30.42				

TARGET	SAMPLE	group	Cq	MEAN
GADPH	Soleus	Control	21.69	21.58
GADPH	Soleus	Control	21.47	
GADPH	Soleus	Control	21.59	
GADPH	Soleus	CPF1	23.25	23.14
GADPH	Soleus	CPF1	23.11	
GADPH	Soleus	CPF1	23.07	
GADPH	Soleus	CPF5	21.03	21.06
GADPH	Soleus	CPF5	21.12	
GADPH	Soleus	CPF5	21.05	

Trial 1:

TARGET	SAMPLE	TREAT	Cq	exp	EXP STE	MEANCq	Cq SEM
alpha	Soleus	Control	28.82	1	0.092616	28.7	0.06119
alpha	Soleus	Control	28.61				
alpha	Soleus	Control	28.69				
alpha	soleus	CPF1	29.82	1.06	0.21	29.78	0.06658
alpha	Soleus	CPF1	29.87				
alpha	Soleus	CPF1	29.65				
alpha	Soleus	CPF5	33.17	0.03	0.32	33.38	0.15213
alpha	Soleus	CPF5	33.31				
alpha	Soleus	CPF5	33.68				

TARGET	SAMPLE	group	Cq	MEAN
GADPH	Soleus	Control	20.36	20.4
GADPH	Soleus	Control	20.44	
GAPDH	Soleus	Control	20.41	
GADPH	Soleus	CPF1	21.69	21.56
GADPH	Soleus	CPF1	21.7	
GADPH	Soleus	CPF1	21.3	
GADPH	Soleus	CPF5	19.98	19.98
GADPH	Soleus	CPF5	19.69	
GADPH	Soleus	CPF5	20.28	

Trial 2:

TARGET	SAMPLE	TREAT	Cq	exp	EXP STE	MEANCq	Cq SEM
alpha	Soleus	Control	28.92	1	0.0082	28.85	0.05568
alpha	Soleus	Control	28.89				
alpha	Soleus	Control	28.74				
alpha	soleus	CPF1	29.59	1.03	0.22	29.71	0.14712
alpha	Soleus	CPF1	30.01				
alpha	Soleus	CPF1	29.55				
alpha	Soleus	CPF5	33.39	0.029	0.07	33.32	0.03283
alpha	Soleus	CPF5	33.31				
alpha	Soleus	CPF5	33.28				

TARGET	SAMPLE	group	Cq	MEAN
GADPH	Soleus	Control	20.58	20.61
GADPH	Soleus	Control	20.61	
GAPDH	Soleus	Control	20.64	
GADPH	Soleus	CPF1	21.45	21.52
GADPH	Soleus	CPF1	21.55	
GADPH	Soleus	CPF1	21.58	
GADPH	Soleus	CPF5	19.95	20.02
GADPH	Soleus	CPF5	20.05	
GADPH	Soleus	CPF5	20.08	

Trial 3:

TARGET	SAMPLE	TREAT	Cq	exp	EXP STE	MEANCq	Cq SEM
Alpha	Soleus	Control	28.87	1	0.011244	28.74	0.06333
Alpha	Soleus	Control	28.71				
Alpha	Soleus	Control	28.66				
Alpha	Soleus	CPF1	30.01	0.94	0.24	29.92	0.04372
Alpha	Soleus	CPF1	29.89				
Alpha	Soleus	CPF1	29.87				
Alpha	Soleus	CPF5	33.37	0.03	0.19	33.32	0.10899
Alpha	Soleus	CPF5	33.49				
Alpha	Soleus	CPF5	33.12				

TARGET	SAMPLE	group	Cq	MEAN
GADPH	Soleus	Control	20.54	20.44
GADPH	Soleus	Control	20.42	
GAPDH	Soleus	Control	20.38	
GADPH	Soleus	CPF1	21.84	21.53
GADPH	Soleus	CPF1	21.47	
GADPH	Soleus	CPF1	21.29	
GADPH	Soleus	CPF5	19.97	19.97
GADPH	Soleus	CPF5	20.12	
GADPH	Soleus	CPF5	19.84	
